# Metabolic dysfunction‐associated steatotic liver disease, insulin sensitivity and continuous glucose monitoring metrics in patients with type 1 diabetes: A multi‐centre cross‐sectional study

**DOI:** 10.1111/dom.16333

**Published:** 2025-03-13

**Authors:** Michela Vergani, Nicolò Diego Borella, Mariangela Rizzo, Matteo Conti, Silvia Perra, Eleonora Bianconi, Elena Sani, Alessandro Csermely, Elisabetta Grespan, Giovanni Targher, Gianluca Perseghin, Alessandro Mantovani, Stefano Ciardullo

**Affiliations:** ^1^ Department of Medicine and Rehabilitation Policlinico di Monza Monza Italy; ^2^ School of Medicine and Surgery University of Milano Bicocca Milan Italy; ^3^ Section of Endocrinology, Diabetes and Metabolism, Department of Medicine University and Azienda Ospedaliera Universitaria Integrata of Verona Verona Italy; ^4^ Department of Medicine University of Verona Verona Italy; ^5^ Metabolic Diseases Research Unit IRCCS Sacro Cuore‐Don Calabria Hospital Negrar di Valpolicella (VR) Italy

**Keywords:** CAP, CMG, eGDR, estimated glucose disposal rate, Fibroscan, hepatic steatosis, insulin resistance, MASLD, metabolic dysfunction‐associated steatotic liver disease, type 1 diabetes

## Abstract

**Background and aim:**

We assessed the prevalence of metabolic dysfunction‐associated steatotic liver disease (MASLD) and significant liver fibrosis in adults with type 1 diabetes mellitus (T1DM) and the association of MASLD with insulin sensitivity and continuous glucose monitoring metrics.

**Methods:**

We consecutively enrolled 198 adults with T1DM undergoing vibration‐controlled transient elastography with liver stiffness measurement (LSM) and controlled attenuation parameter (CAP). All participants had a continuous glucose monitoring (CGM) device. Insulin sensitivity was evaluated by estimated glucose disposal rate (eGDR). MASLD was defined as CAP ≥ 248 db/m and the presence of at least one cardiometabolic risk factor. Significant liver fibrosis was defined as LSM ≥ 7 kPa.

**Results:**

Patients had a mean age of 56 years, mean BMI of 26.0 ± 5.9 kg/m^2^, and mean eGDR of 7.1 ± 2.3 mg/kg/min. 73 (37%) patients had MASLD (using a CAP threshold of 274 dB/m), 16 (8.1%) of whom had significant liver fibrosis. MASLD was associated with a significantly lower eGDR (beta coefficient = −0.367, 95% confidence interval −0.472 to −0.261; *p* < 0.001). This association remained significant, even after adjustment for age, sex, body mass index, plasma triglycerides, diabetes duration, daily insulin dose, time above the range of glucose levels, LSM and chronic kidney disease. No association was observed between MASLD and CGM‐derived metrics. These results were not different when we used a CAP threshold of 274 dB/m for diagnosing MASLD.

**Conclusion:**

In T1DM, MASLD was inversely associated with eGDR and biomarkers of insulin resistance but not with CGM‐derived metrics.

## 1 INTRODUCTION

Metabolic dysfunction‐associated steatotic liver disease (MASLD), formerly termed non‐alcoholic fatty liver disease (NAFLD), is frequently encountered in adult and paediatric populations, with global MASLD prevalence estimates of ~30% and 15%, respectively.^1^, ^2^ To date, MASLD is the most common cause of chronic liver diseases worldwide and is becoming the leading indication for liver transplantation.^3^ The recently proposed change in terminology from NAFLD to MASLD underlines the close interplay between hepatic steatosis and metabolic dysfunction, a condition at the centre of the concept of metabolic syndrome and insulin resistance.^4^ Therefore, it is not surprising that MASLD is very frequent in people with type 2 diabetes mellitus (T2DM), affecting up to ~70%–80% of this patient population.^5^ Notably, patients with T2DM also have a higher risk of progression to more severe forms of MASLD (metabolic dysfunction‐associated steatohepatitis [MASH], advanced fibrosis and cirrhosis), as well as a higher risk of developing adverse cardiovascular and renal outcomes.^6^, ^7^

While T2DM is traditionally viewed as a condition characterized by insulin resistance and relative insulin deficiency, type 1 diabetes mellitus (T1DM) is considered a model of pure insulin deficiency and subsequent hyperglycemia.^8^ Nonetheless, given the rising rates of overweight and obesity in people with T1DM, insulin resistance is frequently encountered in these patients, especially in adulthood.^9^, ^10^ Moreover, insulin replacement therapy occurs in a non‐physiological way because it is administered subcutaneously rather than in the portal circulation. This may lead to relative insulin deficiency in the portal vein and hyperinsulinization of peripheral tissues, thereby promoting insulin resistance by desensitization of the insulin receptor and ectopic fat deposition.^11^ Additionally, patients with T1DM are also exposed to significant plasma glucose fluctuations,^12^ and few patients can achieve good glycaemic control without a significant risk of hypoglycaemia and hyperglycaemia.^13^ Whether and to what extent chronic hyperglycaemia, glycaemic variability and insulin resistance may impact hepatic steatosis and fibrosis in adult patients with T1DM is currently uncertain.

Based on this background of evidence, the two main aims of this multi‐centre, cross‐sectional study, which included adults with T1DM undergoing vibration‐controlled transient elastography (VCTE) with liver stiffness measurement (LSM) and controlled attenuation parameter (CAP) to non‐invasively measure hepatic fibrosis and steatosis, were as follows: (a) to examine the prevalence of MASLD and significant fibrosis; and (b) to assess the association of MASLD with insulin sensitivity, as non‐invasively estimated by glucose disposal rate (eGDR) and continuous glucose monitoring metrics.

## 2 METHODS

### 2.1 Participants

This multi‐centre, cross‐sectional study was conducted at the diabetes outpatient service of the University Hospital of Verona and the diabetes outpatient service of the Policlinico di Monza (Italy). Both local Institutional Ethics Committees approved the study protocol, and written informed consent was obtained from all participants.

In this study, we included 198 adult outpatients with T1DM (56% men; mean age 56 years) who consecutively underwent VCTE and had a continuous glucose monitoring (CGM) device. Of these patients, 95 attended the diabetes outpatient service of the University Hospital of Verona, and 103 attended the diabetes outpatient service of the Policlinico di Monza. The inclusion criteria of the study were as follows: (a) adults (≥18 years) with a diagnosis of T1DM confirmed by undetectable plasma C‐peptide concentrations and the presence of at least one diabetes‐associated autoantibody (GADA, ZnT8A, IAA or IA‐2A) at least 2 years before the study enrolment; (b) patients who had CGM devices and performed a valid VCTE (as discussed below); and (c) patients who signed the informed consent. The exclusion criteria of the study were as follows: (a) patients aged <18 years; (b) subjects with T2DM; (c) pregnant women; (d) a history of significant alcohol consumption (>30 g/day for men and > 20 g/day for women) and other competing causes of hepatic steatosis (e.g., virus and medications); (e) cirrhosis of any aetiology, active cancer or end‐stage renal disease (defined as estimated glomerular filtration rate [eGFR] <15 mL/min/1.73 m^2^ or chronic dialysis).

### 2.2 Clinical and biochemical data

Body mass index (BMI) was measured as kilograms divided by the square of height in meters. Waist circumference was measured at the midpoint between the lowest rib and the iliac crest. Blood pressure was measured with a standard sphygmomanometer after the subject had been seated quietly for at least 5 min. Participants were considered to have arterial hypertension if their blood pressure was ≥140/90 mmHg or if they were taking any anti‐hypertensive agents. At the time of study enrolment, participants were asked to provide estimates of their daily alcohol consumption over the 4 weeks preceding the interview, and the reported alcohol consumption was converted into grams of alcohol (e.g., a 33 cL bottle of beer corresponded to nearly 12 g of absolute alcohol). Moreover, medication use, modalities of insulin administration (i.e., multiple daily injections [MDI] or continuous subcutaneous insulin infusion [CSII]), daily insulin dosages, and information about the type of glucose monitoring device were recorded for all participants. For patients using intermittently scanned continuous glucose monitoring (CGM) devices (i.e., Abbott FreeStyle Libre 2® Glucose Monitoring System) or real‐time CGM devices (i.e., iDexcom G5® CGM System, Dexcom G6® CGM System and Guardian™ 4), the glucose data available from 14 to 90 days preceding the enrolment visit were collected. According to international recommendations, the following short‐term glycaemic control metrics were calculated from CGM data^14^: time in range 70–180 mg/dL (TIR); time below range < 70 mg/dL (TBR); time below range 54–69 mg/dL (low glucose or Level 1 hypoglycaemia) (TBR1); time below range < 54 mg/dL (very low glucose or Level 2 hypoglycaemia) (TBR2); time above range > 180 mg/dL (>10.1 mmol/L) (TAR); time above range 181 to 250 mg/dL (10.1–13.9 mmol/L) (high glucose or Level 1 hyperglycaemia) (TAR1); time above range > 250 mg/dL (>13.9 mmol/L) (very high glucose or Level 2 hyperglycaemia) (TAR2); coefficient of variation (CV). Moreover, the Glycaemia Risk Index (GRI) and its hypoglycaemic and hyperglycaemic components were also calculated as novel CGM metrics.^14^ To ensure adequate CGM data, participants were included in the analysis if at least 70% of expected CGM readings occurring for at least 14 days were available.

Venous blood samples were collected in the morning after an overnight fast in all participants. Haemoglobin A1c (HbA1c), creatinine, lipids and other biochemical parameters were measured at the Central Laboratory of the University Hospital of Verona and the Policlinico di Monza by standard laboratory procedures. Low‐density lipoprotein (LDL)‐cholesterol was calculated using Friedewald's equation. HbA1c levels were measured using the high‐performance liquid chromatography analyser Tosoh‐G7 (Tosoh Bioscience Inc., Tokyo, Japan). The glomerular filtration rate (eGFR) was estimated using the Chronic Kidney Disease Epidemiology Collaboration (CKD‐EPI) equation.^15^ Urinary albumin excretion rate was assessed with an immuno‐nephelometric assay (Beckman Coulter IMMAGE; Beckman Coulter Instruments, Fullerton, CA, USA) on a morning spot urine sample and expressed as the albumin‐to‐creatinine ratio (ACR).^16^ Microalbuminuria was defined as a urinary ACR ranging from 30 to 300 mg/g creatinine. Macroalbuminuria was defined as a urinary ACR ≥300 mg/g creatinine.^16^ In this multi‐centre study, chronic kidney disease (CKD) was defined as the presence of eGFR_CKD‐EPI_ <60 mL/min/1.73 mm^2^ and/or macroalbuminuria.

The presence of ischemic heart disease was defined as a documented history of myocardial infarction, angina or coronary revascularization procedures. The presence of ischemic stroke was defined by a documented history. The presence of diabetic retinopathy, diagnosed with fundoscopy after pupillary dilation, was also recorded in all participants.

### 2.3 Estimated glucose disposal rate

eGDR (expressed as mg/kg/min) was used as a non‐invasive measure of insulin sensitivity and was calculated as follows^17^: eGDR = 21.158 – (0.09*waist circumference [cm]) – (3.407 × hypertension [1 = yes; 0 = no]) – (0.551*HbA1c [%]).

### 2.4 Vibration‐controlled transient elastography (VCTE)

In both participating centres, VCTE with liver stiffness measurement (LSM) and controlled attenuation parameter (CAP) (i.e., FibroScan® Compact 530) was performed by trained operators, who were blinded to the clinical and biochemical data of participants, on the right lobe of the liver through intercostal spaces on individuals lying in the decubitus position with the right arm in abduction.^18^ The success rate was calculated as the number of successful measurements divided by the total number of acquisitions. The median value of the successful LSM was expressed in kilopascal (kPa), whereas the median value of the successful CAP score was expressed in decibels per meter (db/m).^18^ Only LSM and CAP score data, acquired from at least 10 successful measurements, a success rate of 100% and IQR/median for both LSM and CAP score of less than 0.30, were considered reliable.^18^ MASLD was defined as CAP ≥248 db/m and the presence of at least one common cardiometabolic risk factor, typically used for diagnosing metabolic syndrome.^19^, ^20^ Significant liver fibrosis was defined as LSM ≥7 kPa.^19^ As a sensitivity analysis, we also repeated all statistical analyses using a CAP threshold of 274 dB/m for diagnosing hepatic steatosis, according to Eddowes et al^21^

### 2.5 Statistical analysis

Continuous variables were expressed as means ± SD or medians and inter‐quartile ranges (IQRs), whereas categorical variables were expressed as relative percentages. Differences in the baseline clinical and biochemical characteristics of participants stratified by MASLD status were tested using the chi‐squared test for categorical variables, the Student's *t*‐test for normally distributed continuous variables and the Mann–Whitney test for non‐normally distributed variables. Differences in the baseline clinical and biochemical characteristics of participants stratified by eGDR tertiles were tested by the chi‐squared test for categorical variables, the one‐way ANOVA for normally distributed continuous variables, and the Kruskal–Wallis test for non‐normally distributed variables. Spearman's rank correlation analysis was used to assess the univariable association between CAP and eGDR values. To evaluate the independent association between MASLD and eGDR, we used linear regression models in which eGDR (logarithmically transformed before the analysis) was the dependent variable of all regression models. Specifically, we performed two linear regression models. The first model was unadjusted; the second model was adjusted for age, sex, BMI, triglycerides, diabetes duration, time above range (TAR), daily insulin dose, presence of CKD and LSM. In these two regression models, we included as covariates both the variables that significantly differed between the presence/absence of MASLD, eGDR tertiles and those based on potential biological plausibility. We did not also adjust for waist circumference, HbA1c and hypertension because these covariates are included in the eGDR formula. All analyses were also repeated using a CAP threshold of 274 dB/m^21^ for diagnosing MASLD. We did not re‐run such analyses stratified by the presence/absence of significant fibrosis, given the small number of patients with LSM ≥7 kPa.

A *p*‐value <0.05 was considered statistically significant. All statistical analyses were performed using Python 3.12.7 (https://www.python.org) and the following packages: *pandas* (version 2.2.3), *numpy* (version 1.24), *matplotlib* (version 3.9), *seaborn* (version 0.13.2), *statsmodels* (version 0.15), *researchpy* (version 0.3.6) and *scipy* (version 1.14.1).

## 3 RESULTS

Of the 198 consecutive outpatients with T1DM included in the study (56% men, mean age 56 years, median diabetes duration 26 years [IQR 14–39 years], mean BMI 26.0 ± 5.9 kg/m^2^, mean HbA1c 7.5 ± 1.2% and mean eGDR 7.1 ± 2.3 mg/kg/min), 182 patients were treated with multiple daily injections (MDI), whereas 16 were treated with continuous subcutaneous insulin infusion (CSII). All participants used a continuous glucose monitoring (CGM) device. In addition, 73 (37%) patients had MASLD (defined as CAP ≥248 db/m and coexistence of at least one cardiometabolic risk factor), of whom 16 (8.1%) patients had significant liver fibrosis (LSM ≥7 kPa). Using a CAP threshold of 274 dB/m for diagnosing MASLD, we found that 41 (22.5%) patients had MASLD, of whom 6 (14.6%) had significant liver fibrosis (LSM ≥7 kPa).

The main clinical and biochemical characteristics of participants, stratified by MASLD status (using a CAP threshold of 248 dB/m), are reported in Table 1. Compared with those without MASLD, patients with MASLD were more likely to be men, centrally overweight or obese, and had higher plasma triglycerides, a greater daily insulin dose and lower plasma HDL cholesterol. Patients with MASLD also had a higher prevalence of hypertension and were more frequently treated with metformin, angiotensin‐converting enzyme inhibitors (ACEIs) or angiotensin II receptor antagonists (ARBs). Conversely, age, duration of diabetes, HbA1c, kidney function parameters, alanine transaminase, LSM measurements, as well as the prevalence of diabetic retinopathy, ischaemic heart disease and ischaemic stroke, and the use of anti‐platelets, statins and anti‐hypertensive agents (except for ACEIs/ARBs) did not differ significantly between the two groups. Notably, the short‐term glycaemic control metrics, as assessed by CGM, were also not different between the two groups.

**TABLE 1:** Clinical and biochemical characteristics of adult patients with type 1 diabetes stratified by MASLD status, evaluated by vibration‐controlled transient elastography (VCTE) with controlled attenuation parameter (using a CAP threshold of 248 dB/m).

	No MASLD (*n* = 125)	MASLD (*n* = 73)	*p* values
Age (years)	55 ± 11	57 ± 11	0.151
Men (%)	47.2	73.9	**<0.001**
Diabetes duration (years)	24 (12–38)	30 (20–41)	0.062
Body mass index (kg/m^2^)	24.4 ± 6.4	28.8 ± 3.7	**<0.001**
Waist circumference (cm)	87.2 ± 11.7	103 ± 11.6	**<0.001**
Obesity (%)	7.2	35.6	**<0.001**
Systolic blood pressure (mmHg)	131 ± 19	136 ± 18	0.087
Diastolic blood pressure (mmHg)	77 ± 11	79 ± 10	0.228
HbA1c (%)	7.5 ± 1.0	7.5 ± 1.4	0.906
Total cholesterol (mg/dL)	162 ± 31	158 ± 46	0.458
HDL‐cholesterol (mg/dL)	62 ± 14	54 ± 14	**<0.001**
Triglycerides (mg/dL)	69 (52–85)	80 (64–94)	**0.001**
Creatinine (mg/dL)	0.86 ± 0.28	0.86 ± 0.20	0.935
eGFR_CKD‐EPI_ (mL/min/1.73 m^2^)	79.3 ± 20.0	75.8 ± 16.9	0.215
AST (IU/L)	22 (18–26)	23 (19–28)	0.233
Liver stiffness (kPa)	4.7 (3.8–5.8)	5.1 (4.3–5.9)	0.088
CKD (%)	12.8	21.9	0.093
Microalbuminuria (%)	6.5	11.4	0.233
Macroalbuminuria (%)	4.1	1.0	0.087
Diabetic retinopathy (%)	32.8	39.7	0.325
Hypertension (%)	38.4	56.2	**0.015**
Ischemic heart disease (%)	2.4	8.2	0.058
Ischemic stroke (%)	0.8	1.4	0.698
Daily insulin dose (IU/Kg/day)	0.56 ± 0.19	0.69 ± 0.31	**<0.001**
Metformin (%)	5.6	17.8	**0.006**
Anti‐platelets (%)	18.4	23.3	0.409
ACE‐inhibitors/ARBs (%)	26.4	39.7	0.051
Beta blockers (%)	9.6	13.7	0.376
Calcium‐channel blockers (%)	12.8	12.3	0.923
Diuretics (%)	6.4	13.7	0.085
Statins (%)	48.0	56.2	0.268
*CGM metrics*			
TIR 70–180 mg/dL	59.1 ± 17.7	58.8 ± 13.9	0.892
TBR	2.9 ± 3.3	2.8 ± 3.3	0.965
TBR1	2.5 ± 2.8	2.4 ± 2.6	0.761
TBR2	0.3 ± 0.8	0.4 ± 0.8	0.451
TAR	38.1 ± 18.6	37.5 ± 14.7	0.829
TAR1	23.9 ± 8.8	26.5 ± 9.6	0.052
TAR2	12.8 ± 12.4	11.1 ± 8.3	0.282
GRI	46.6 ± 22.6	45.8 ± 16.8	0.802
GRI hypoglycaemia component	6.9 ± 8.2	6.9 ± 8.2	0.971
GRI hyperglycaemia component	39.7 ± 23.4	38.9 ± 16.7	0.818
%CV	36.0 ± 5.0	35.7 ± 5.1	0.703

*Note*: Sample size, *n* = 198. Data are expressed as means ± SD, medians and interquartile ranges (IQRs) or percentages. Differences among the two patient groups were tested by the Chi‐squared test for categorical variables, the Student's *t* test for normally distributed continuous variables and the Mann–Whitney *U* test for non‐normally distributed variables (i.e., diabetes duration, triglycerides, AST, liver stiffness measurement). CKD was defined as eGFR_CKD‐EPI_ < 60 mL/min/1.73 m^2^ and/or macroalbuminuria. MASLD was defined as CAP ≥248 db/m and the presence of at least one common cardiometabolic risk factor.

Abbreviations: ACE, angiotensin‐converting‐enzyme inhibitor; ARB, angiotensin II receptor blocker; AST, aspartate aminotransferase; BMI, body mass index; CGM, continuous glucose monitoring; CKD, chronic kidney disease; eGFR_CKD‐EPI_, estimated glomerular filtration rate calculated by the CKD‐Epidemiology Collaboration study equation. CV, Coefficient of variation; GRI, Glycaemia Risk Index; MASLD, metabolic dysfunction‐associated steatotic liver disease; TAR, Time above range > 180 mg/dL (>10.1 mmol/L); TAR1, Time above range 181–250 mg/dL (10.1–13.9 mmol/L) (high glucose or Level 1 hyperglycaemia); TAR2, Time above range > 250 mg/dL (>13.9 mmol/L) (very high glucose or Level 2 hyperglycaemia); TIR, Time in range 70–180 mg/dL (3.9–10.0 mmol/L); TBR, Time below range < 70 mg/dL (<3.9 mmol/L); TBR1, Time below range 54–69 mg/dL (3.0–3.9 mmol/L) (low glucose or Level 1 hypoglycaemia); TBR2, Time below range < 54 mg/dL (<3.0 mmol/L) (very low glucose or Level 2 hypoglycaemia).

Table S1 shows the main clinical and biochemical characteristics of participants, stratified by overweight/obese status. Patients with overweight/obesity were more likely to be men, had a longer disease duration, higher values of waist circumference, triglycerides, LSM, daily insulin dose, as well as lower plasma HDL cholesterol compared with those with normal body weight. In addition, the former also had a significantly higher prevalence of MASLD. The short‐term glycaemic control metrics (as assessed by CGM) did not differ between the two groups, apart from TIR.

**FIGURE 1:**
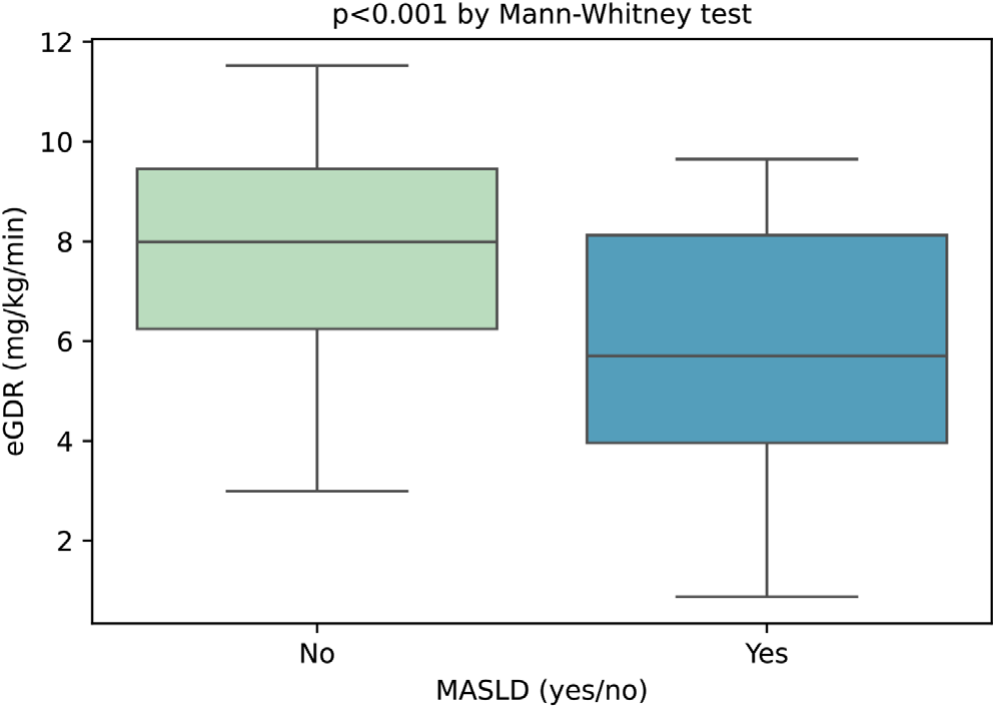
Boxplots of the estimated glucose disposal rate (eGDR) in adult patients with T1DM stratified by MASLD status, evaluated by vibration‐controlled transient elastography (VCTE) with controlled attenuation parameter (using a CAP threshold of 248 dB/m).

Figure 1 shows the boxplots of the eGDR in patients stratified by MASLD status (using a CAP threshold of 248 dB/m). Patients with MASLD had a significantly lower eGDR than those without MASLD (5.71 [IQR 3.96–8.12] mg/kg/min in the MASLD group vs. 7.99 [IQR 6.24–9.45] mg/kg/min in the non‐MASLD group; *p* < 0.001 by Mann–Whitney test). Similar results were also observed when we used a CAP threshold of 274 dB/m (5.42 [IQR 3.59–7.73] mg/kg/min in the MASLD group vs. 7.88 [IQR 5.85–7.88] mg/kg/min in the non‐MASLD group; *p* < 0.001) (Figure S1).

Table 2 summarizes the main clinical and biochemical characteristics of participants, stratified by eGDR tertiles. Compared with those in the second and third tertiles of eGDR, patients in the first eGDR tertile (i.e., subjects more insulin resistant) were more likely to be older, centrally overweight or obese, had a longer diabetes duration, higher values of systolic blood pressure, HbA1c, total cholesterol, LSM and daily insulin doses, and lower values of HDL cholesterol and eGFR. The prevalence of MASLD, CKD, diabetic retinopathy, hypertension, as well as the use of metformin, anti‐platelets, statins and anti‐hypertensive medications were higher in patients in the 1st tertile of eGDR than in those in the 2nd and 3rd eGDR tertiles. Conversely, the short‐term glycaemic control metrics did not differ significantly among the three patient groups.

**TABLE 2:** Clinical and biochemical characteristics of adult patients with type 1 diabetes stratified by estimated glucose disposal rate (eGDR) tertiles.

	1st eGDR tertile (*n* = 66) (4.4 ± 1.2 mg/kg/min)	2nd eGDR tertile (*n* = 66) (7.4 ± 0.7 mg/kg/min)	3rd eGDR tertile (*N* = 66) (9.5 ± 0.8 mg/kg/min)	*p* values
Age (years)	62 ± 10	55 ± 10	51 ± 11	**<0.001**
Men (%)	68.2	54.6	48.5	0.065
Diabetes duration (years)	30 (22–39)	29 (16–40)	23 (9–34)	**0.027**
Body mass index (Kg/m^2^)	28.5 ± 8.3	25.6 ± 4.0	23.9 ± 3.3	**<0.001**
Waist circumference (cm)	102 ± 13	92 ± 13	85 ± 9	**<0.001**
Obesity (%)	28.8	19.7	4.6	**0.001**
Systolic blood pressure (mmHg)	136 ± 19	132 ± 20	127 ± 15	**0.007**
Diastolic blood pressure (mmHg)	77 ± 11	79 ± 10	79 ± 10	0.408
HbA1c (%)	7.9 ± 1.0	7.5 ± 1.5	7.2 ± 0.8	**0.006**
Total cholesterol (mg/dL)	152 ± 41	159 ± 36	170 ± 32	**0.016**
HDL‐cholesterol (mg/dL)	55 ± 13	60 ± 16	62 ± 15	**0.021**
Triglycerides (mg/dL)	73 (63–89)	72 (53–85)	70 (52–91)	0.372
Creatinine (mg/dL)	0.89 ± 0.28	0.88 ± 29	0.81 ± 0.15	0.149
eGFR_CKD‐EPI_ (mL/min/1.73 m^2^)	73.1 ± 19.3	77.4 ± 20.3	83.7 ± 15.9	**0.005**
AST (IU/L)	21 (18–26)	23 (20–27)	23 (18–26)	0.438
MASLD (%)	63.6	27.3	19.7	**<0.001**
Liver stiffness (kPa)	5.2 (4.3–6.0)	4.8 (3.6–5.9)	4.6 (3.9–5.5)	**0.048**
CKD (%)	22.7	21.2	4.6	**0.007**
Microalbuminuria (%)	10.8	8.1	6.1	0.618
Macroalbuminuria (%)	4.6	3.2	0	0.233
Diabetic retinopathy (%)	43.9	40.9	21.2	**0.012**
Hypertension (%)	93.9	39.4	1.5	**<0.001**
Ischemic heart disease (%)	9.1	3.0	1.5	0.087
Ischemic stroke (%)	1.5	1.5	0	0.603
Daily insulin dose (IU/Kg/day)	0.73 ± 0.31	0.58 ± 0.21	0.50 ± 0.14	**<0.001**
Metformin (%)	13.6	7.6	9.1	0.485
Anti‐platelets (%)	34.9	16.7	9.1	**<0.005**
ACE‐inhibitors/ARBs (%)	57.6	27.3	9.1	**<0.001**
Beta blockers (%)	21.2	9.1	3.0	**0.003**
Calcium antagonists (%)	18.2	16.7	3.0	**0.016**
Diuretics (%)	16.7	9.1	1.5	**0.010**
Statins (%)	69.7	45.5	37.9	**<0.005**
CGM metrics				
TIR 70–180 mg/dL	56.4 ± 16.3	58.4 ± 16.1	62.1 ± 16.6	0.129
TBR	2.8 ± 3.3	3.2 ± 3.8	2.6 ± 2.8	0.605
TBR1	2.3 ± 2.7	2.8 ± 3.1	2.4 ± 2.4	0.592
TBR2	0.37 ± 0.80	0.33 ± 0.94	0.23 ± 0.44	0.566
TAR	39.8 ± 17.4	38.4 ± 17.1	35.3 ± 17.1	0.296
TAR1	26.8 ± 10.8	24.6 ± 7.6	23.3 ± 8.6	0.088
TAR2	12.5 ± 11.2	12.9 ± 11.6	11.1 ± 10.5	0.597
GRI	48.1 ± 19.9	48.0 ± 21.0	42.7 ± 20.8	0.229
GRI hypoglycaemia component	6.7 ± 8.2	7.6 ± 9.5	6.4 ± 6.8	0.652
GRI hyperglycaemia component	41.5 ± 20.4	40.4 ± 21.9	36.4 ± 20.9	0.344
%CV	35.9 ± 4.9	36.3 ± 5.2	35.5 ± 5.1	0.631

*Note*: Sample size, n = 198. Data are expressed as means ± SD, medians and interquartile ranges (IQRs) or relative percentages. Differences among the three patient groups were tested by the Chi‐squared test for categorical variables, the one‐way ANOVA for normally distributed continuous variables and the Kruskal–Wallis test for non‐normally distributed variables (i.e., diabetes duration, triglycerides, AST, liver stiffness measurement). CKD was defined as eGFR_CKD‐EPI_ < 60 mL/min/1.73 m^2^ and/or macroalbuminuria. MASLD was defined as CAP ≥248 db/m and presence of at least one cardiometabolic risk factor.

Abbreviations: ACE, angiotensin‐converting enzyme inhibitor; ARB, angiotensin II receptor blocker; AST, aspartate aminotransferase; BMI, body mass index; CGM, continuous glucose monitoring; CKD, chronic kidney disease; eGFR_CKD‐EPI_, estimated glomerular filtration rate calculated by the CKD‐Epidemiology Collaboration study equation. CV, Coefficient of variation; GRI, Glycaemia Risk Index; MASLD, metabolic dysfunction‐associated steatotic liver disease; TAR, Time above range > 180 mg/dL (>10.1 mmol/L); TAR1, Time above range 181–250 mg/dL (10.1–13.9 mmol/L) (high glucose or Level 1 hyperglycaemia); TAR2, Time above range > 250 mg/dL (>13.9 mmol/L) (very high glucose or Level 2 hyperglycaemia); TIR, Time in range 70–180 mg/dL (3.9–10.0 mmol/L); TBR, Time below range < 70 mg/dL (<3.9 mmol/L); TBR1, Time below range 54–69 mg/dL (3.0–3.9 mmol/L) (low glucose or Level 1 hypoglycaemia); TBR2, Time below range < 54 mg/dL (<3.0 mmol/L) (very low glucose or Level 2 hypoglycaemia.

**FIGURE 2:**
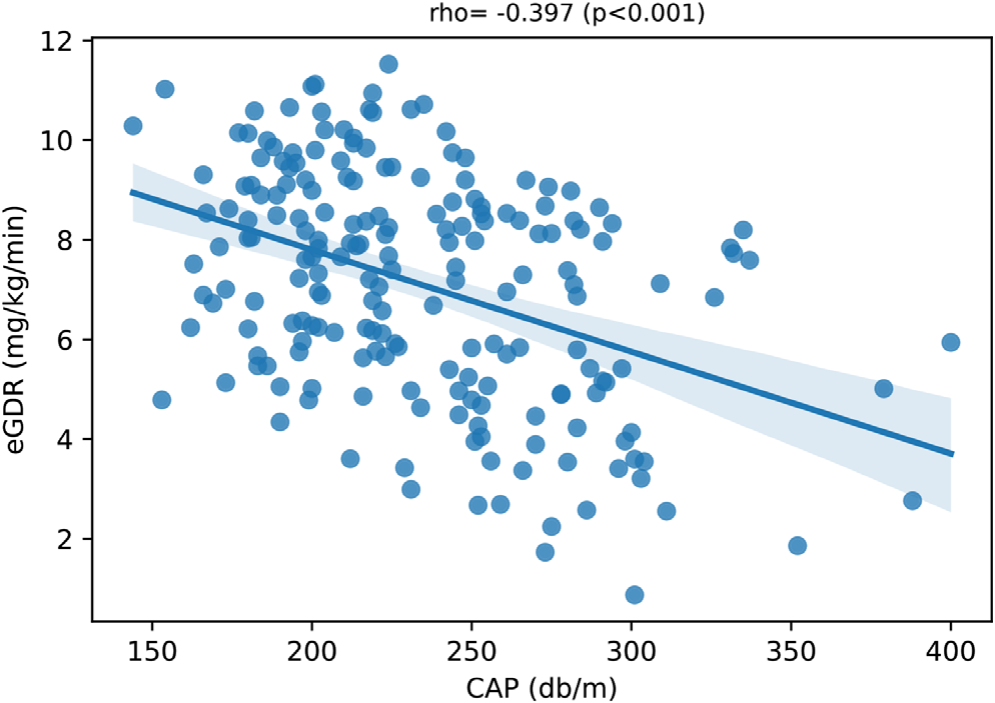
Univariate Spearman's rank correlation between estimated glucose disposal rate (eGDR) (on Y axis) and controlled attenuation parameter (CAP) (on X axis) in patients with T1DM (Spearman's rho coefficient = −0.397, *p* < 0.001).

Figure 2 shows a scatterplot of the linear correlation between CAP (on the X‐axis) and eGDR values (on the Y‐axis). CAP levels were inversely associated with eGDR (Spearman's rho coefficient: −0.397, *p* < 0.001).

Table 3 shows the association between MASLD (using a CAP threshold of 248 dB/m) and eGDR, evaluated by univariable and multivariable linear regression models. In the unadjusted model, MASLD was associated with a significantly lower eGDR (beta coefficient = −0.367, 95% CI −0.472 to −0.261; *p* < 0.001). Notably, this association remained significant even after adjustment for age, sex, diabetes duration, BMI, plasma triglycerides, CKD, LSM, time above the range (TAR) of glucose levels and daily insulin dose (adjusted model 1: beta coefficient = −0.223, 95% CI −0.324 to −0.121; *p* < 0.001). Other variables independently associated with eGDR were older age, higher BMI, higher plasma triglycerides and greater daily insulin dose.

**TABLE 3:** Linear regression analyses—Association between estimated glucose disposal rate (eGDR) and MASLD, using a CAP threshold of 248 dB/m, in adult patients with type 1 diabetes.

	Beta coefficients	95% confidence intervals	*p* values
**Unadjusted model**			
MASLD (yes vs. no)	−0.359	−0.464 to −0.254	**<0.001**
**Adjusted model 1**			
MASLD (yes vs. no)	−0.223	−0.324 to −0.121	**<0.001**
Age (years)	−0.010	−0.015 to −0.007	**<0.001**
Sex (men vs. women)	−0.040	−0.136 to 0.056	0.412
Body mass index (kg/m^2^)	−0.013	−0.021 to −0.005	**0.002**
Triglycerides (mg/dL) (Log Scale)	−0.131	−0.025 to 0.236	**0.015**
Time above range (181–250 mg/dL) (TAR) (Log Scale)	−0.063	−0.138 to 0.012	0.098
Diabetes duration (years) (Log Scale)	0.001	−0.003 to 0.003	0.974
Daily insulin dose (IU/Kg/day)	−0.458	−0.654 to −0.261	**<0.001**
CKD (yes vs. no)	−0.012	−0.140 to 0.116	0.851
Liver stiffness (kPa) (Log Scale)	−0.083	−0.223 to 0.058	0.248

*Note*: Sample size, n = 198. Data are expressed as beta coefficients and 95% confidence intervals estimated by linear regression analyses. The dependent variable of all models was the estimated glucose disposal rate (eGDR) (logarithmically transformed before the analyses). MASLD (metabolic dysfunction‐associated steatotic liver disease) was defined as CAP ≥248 db/m and the presence of at least one cardiometabolic risk factor. CKD (chronic kidney disease) was defined as eGFR_CKD‐EPI_ < 60 mL/min/1.73 m^2^ and/or macroalbuminuria.

Almost identical results to those reported in Table 3 above were observed when we repeated the analyses using a CAP threshold of 274 dB/m for diagnosing MASLD (Table S3).

Figure S2 shows the percentages of daily insulin dose tertiles by MASLD status, diagnosed using either a CAP threshold of 248 dB/m (panel A) or a CAP threshold of 274 dB/m (panel B). In patients with MASLD (using a CAP threshold of 248 dB/m), there were more patients in the 3rd tertile of daily insulin dose than those with no MASLD (panel A). Very similar results were found even when MASLD was diagnosed using a CAP threshold of 274 dB/m (panel B).

## 4 DISCUSSION

In this multi‐centre, cross‐sectional study, which included 198 middle‐aged Italian individuals with established T1DM, we reported three relevant observations. First, the prevalence of MASLD, assessed by VCTE (FibroScan® Compact 530) using a CAP threshold of 248 dB/m, was nearly 37% in this patient population. The observed prevalence of MASLD was slightly higher than that reported in a meta‐analysis of 20 observational studies with 3910 adult patients with T1DM.^22^ In that meta‐analysis, De Vries et al. reported that the pooled prevalence of MASLD (as detected by liver ultrasonography, transient elastography, magnetic resonance imaging or liver biopsy) was 22% (95% CI 13.9%–31.2%), as also further confirmed in a subsequent meta‐analysis by Souza et al. reporting a pooled prevalence of MASLD of 22.2% (95% CI 15.6%–30.6%) in people with T1DM.^22^, ^23^ In our opinion, the difference in the prevalence of MASLD reported in our study and in these two meta‐analyses might be partly explained by clinical and biochemical characteristics of our T1DM patients in terms of age (mean 56 ± 11 years), diabetes duration (median 26 years) and prevalence of obesity (~18%). Moreover, in the meta‐analysis by De Vries et al.^22^ the included studies used different methods to assess MASLD, while we exclusively used VCTE with LSM and CAP. Of note, the CAP cut‐offs used might even reveal different prevalence rates within the same population. In fact, in our study, using a CAP threshold of 248 dB/m as proposed by Karlas et al.,^19^ we found that the prevalence of MASLD was higher than the prevalence of MASLD when we used a CAP cut‐off of 274 dB/m(37% vs. 22.5%), as proposed by Eddowes et al.^21^ This finding highlights the need to find CAP cut‐offs specific to age, sex and other characteristics of the population being studied (e.g., non‐diabetes vs. T2DM vs. T1DM). Second, in our study, we found that the prevalence of significant liver fibrosis (defined as LSM ≥7 kPa) was approximately 8%. At present, few studies are using VCTE to non‐invasively assess liver fibrosis in people with T1DM. A recent meta‐analysis by Ciardullo et al. that included three observational studies for a total of 390 adult patients with T1DM reported a pooled prevalence of significant fibrosis of 5.2%.^24^ That said, the rates of hepatic steatosis and fibrosis are significantly lower in adult patients with T1DM than in those with T2DM.^25^ Third, in this study, we also found a significant inverse association between CAP‐defined hepatic steatosis and eGDR. Notably, this association remained significant even after adjustment for known predictors of insulin resistance, such as age, sex, diabetes duration, BMI, plasma triglycerides, CKD, LSM and glycaemic control, including daily insulin dose. The association between hepatic steatosis and insulin resistance is well known, and it has been demonstrated in different patient populations using various diagnostic methods. Nonetheless, this aspect was seldom described in patients with T1DM. The eGDR is a simple and non‐invasive measure of insulin sensitivity based on waist circumference, hypertension and HbA1c, which can also be used in people with T1DM.^26^ eGDR is a reliable non‐invasive proxy of insulin sensitivity compared with euglycaemic hyperinsulinemic clamp and is suitable for clinical practice and cohort studies.^17^ Interestingly, many observational studies reported an inverse association between eGDR and the risk of hard clinical outcomes. For instance, Garofolo et al. reported a strong inverse relationship of eGDR with adverse cardiovascular outcomes and all‐cause mortality in people with T1DM.^27^ Given the inverse association between MASLD and eGDR identified in our study, as well as the evidence supporting a strong association between MASLD and the risk of cardiovascular events in patients with T1DM,^28^ it is likely that the association we observed between MASLD and eGDR is, at least in part, mediated by some metabolic syndrome features. Indeed, as reported in Table S1, patients with T1DM and overweight/obesity had a greater central fat distribution and plasma lipid profiles comparable to those seen in metabolic syndrome. Giving further support to this hypothesis, in a recent observational study including 150 patients with T1DM and 100 patients with T2DM, in which VCTE was used to detect MASLD, De Vries et al.^29^ reported that both in patients with T1DM and in those with T2DM, waist circumference, BMI and metabolic syndrome were positively associated with MASLD while estimated insulin sensitivity (eGDR) was negatively associated with MASLD, even after adjustment for age, sex and diabetes duration. These investigators also showed that predictors of MASLD were similar between patients with T1DM and those with T2DM. Indeed, data suggest that insulin resistance may be the major driver of the disease in both conditions. The increased free fatty acid release from partially suppressed adipose tissue lipolysis leads to hepatic fat accumulation.^30^ According to the “multiple hit hypothesis”, several insults (such as oxidative stress and low‐grade inflammation) are involved in the pathophysiology of MASLD and can facilitate the progression from simple steatosis to MASH and cirrhosis.^31^ Differences in the prevalence of MASLD and liver fibrosis between patients with T1DM and T2DM might be partly related to differences in baseline characteristics of included populations (e.g., age) but also to the fact that almost all patients with T2DM are insulin resistant and have been exposed to insulin resistance for many years even before diabetes onset. Conversely, insulin resistance in people with T1DM is often secondary to non‐physiologic exogenous insulin exposure and changes in the anthropometric features during the course of the lifetime in parallel with aging. In this regard, we also observed more T1DM patients in the third tertile of daily insulin dose among those with MASLD compared with their counterparts without MASLD.

However, it is also important to note that in our study, the inverse association between eGDR and MASLD remained significant even after adjustment for age, sex, BMI, triglycerides, diabetes duration, glycaemic control, CKD and LSM. This finding might indicate that, on the one hand, MASLD worsens insulin resistance and, on the other hand, insulin resistance, rather than chronic hyperglycaemia, is a risk factor for hepatic fat accumulation in patients with T1DM. This may also be partly supported by the lack of an association between MASLD and CGM‐derived metrics we observed in our study, which is in line with the recent findings by Fuhri Snethlage et al^32^ However, it is also important to note that in a recent study, Aernouts et al. reported a significant association between MASLD and CGM‐derived metrics in 302 adult patients with T1DM.^33^ This study differed from our study in the methods used for diagnosing MASLD (ultrasound rather than VCTE) and in the prevalence of MASLD. Furthermore, Aernouts et al. found a significant association between TIR, TAR and MASLD in the multivariable regression model, but no difference in the prevalence of MASLD was observed when patients were stratified according to TIR below or above 70%. These findings suggest that if an association exists, it is probably weak. In another observational study of 244 children and adolescents with T1DM, our group recently reported that the mean HbA1c values from diabetes onset and TAR were the independent predictors of MASLD, as detected by ultrasonography.^34^ Speculatively, we believe that the differences in the association of CGM‐derived metrics with MASLD among adults and adolescents with T1DM might be due to some factors, such as diabetes duration, prevalence of MASLD, prevalence of overweight/obesity and time of exposure to insulin therapy.

An important issue that still needs to be addressed is related to the potential predictors of liver fibrosis in adult patients with T1DM. The available observational studies, including the present one, are probably not powered enough for this specific analysis. However, in our univariate analysis (Table 2), we observed that patients in the 1st eGDR tertile had higher values of LSM than those in the 2nd and 3rd eGDR tertiles. That said, we believe that future endeavours are needed to fully account for this knowledge gap, especially because the severity of liver fibrosis represents the histological feature more strongly associated with the risk of liver‐related and extra‐hepatic complications in MASLD.^35^

Our study has some important limitations that should be mentioned. First, the observational design did not allow us to establish a cause–effect relationship between MASLD and eGDR in people with T1DM. Second, our study included a cohort of Italian adults with T1DM with relatively good glycaemic control who regularly attended the diabetes outpatient services and accepted to undergo VCTE with LSM and CAP. Hence, these results might not be generalizable to other patient groups. In addition, a control group of non‐diabetic individuals was lacking. Third, although VCTE is considered a well‐performing technique to non‐invasively measure the degree of liver fibrosis and steatosis, it is recommended as a second‐step imaging technique and is not the “gold standard” technique (which remains the liver biopsy).^20^ That said, none of our patients with T1DM were candidates for liver biopsy, as they had fairly normal serum liver enzyme levels and did not satisfy the criteria established by current guidelines.^20^ Similarly, the *“gold standard”* technique for assessment of insulin sensitivity (i.e., the euglycaemic hyperinsulinemic clamp) was not performed in our study, as this technique is invasive, time‐consuming and not recommended in clinical practice. Although not in all studies,^36^ eGDR showed good performance compared with the euglycaemic clamp technique.^26^ However, because eGDR is a calculated index and not a direct measure of insulin sensitivity, the impact of each variable included in the eGDR formula on the association with MASLD might differ in various cohorts and might even be influenced by several factors, such as genetics, physical activity, medication use and body composition. It is also complicated to separate insulin resistance from various associated factors, such as central adiposity and hypertension. In this sense, for instance, hypertension often coexists with insulin resistance and is also strongly related to MASLD.^37^ Hence, these aspects should be considered when interpreting the results of our study. That said, using eGDR can offer some advantages, including the use of a simple, non‐invasive and cost‐effective method, which can be reliably used in epidemiological research and routine clinical practice to assess individuals with T1DM at higher risk for insulin resistance‐related complications.^38^, ^39^, ^40^

Notwithstanding these limitations, our study also has important strengths. First, this is a multi‐centre study. Second, in both centres, we used the same technique (i.e., FibroScan® Compact 530) to assess liver steatosis and fibrosis. Third, we excluded patients with important comorbidities (such as cirrhosis, advanced renal disease or active cancer), as we believe that the inclusion of patients with such comorbidities might have confounded the interpretation of data.

In conclusion, this multi‐centre, cross‐sectional study shows a relatively high prevalence of MASLD (as assessed by VCTE with CAP) in adults with a long‐lasting duration of T1DM. In addition, there is a significant and inverse association between MASLD and eGDR (a reliable estimate of insulin sensitivity), even after adjustment for age, sex, diabetes duration, BMI, CKD, plasma triglycerides, time above the range (TAR) of glucose levels and daily insulin dose. Conversely, CGM‐derived metrics were not significantly associated with MASLD. Further studies are needed to corroborate these findings in other independent samples of adults with T1DM.

## AUTHOR CONTRIBUTIONS

S.C. and A.M. developed the study design, researched the data, contributed to discussions, wrote the manuscript and edited the manuscript. All Authors planned and supervised this research, researched the data, contributed to discussions and edited the manuscript. G.T. and G.P. revised the manuscript for important intellectual content. All authors read and approved the final manuscript. S.C. and A.M. are the guarantors of this work.

## FUNDING INFORMATION

We received no specific funding for the present study.

## CONFLICT OF INTEREST STATEMENT

There were no conflicts of interest relevant to this report.

### PEER REVIEW

The peer review history for this article is available at https://www.webofscience.com/api/gateway/wos/peer-review/10.1111/dom.16333.

## Supporting information


**FIGURE S1.** Boxplots of the estimated glucose disposal rate (eGDR) in adult patients with T1DM stratified by MASLD status, evaluated by vibration‐controlled transient elastography (VCTE) with controlled attenuation parameter (using a CAP threshold of 274 dB/m).
**FIGURE S2.** Percentages of daily insulin dose tertiles by MASLD status, diagnosed using either a CAP threshold of 248 dB/m (Panel A) or a CAP threshold of 274 dB/m (Panel B).
**TABLE S1.** Clinical and biochemical characteristics of adult patients with type 1 diabetes stratified by overweight/obesity status.
**TABLE S2.** Univariable and multivariable linear regression analyses – Association between estimated glucose disposal rate (eGDR) and MASLD, using a CAP threshold of 274 dB/m, in adult patients with type 1 diabetes.

## Data Availability

The data that support the findings of this study are available on request from the corresponding author. The data are not publicly available due to privacy or ethical restrictions.
