# Supplementary material for: Metabolic dysfunction‐associated steatotic liver disease, insulin sensitivity and continuous glucose monitoring metrics in patients with type 1 diabetes: A multi‐centre cross‐sectional study
Source: Diabetes Obes Metab. 2025 Mar 13;27(6):3201–11. doi: 10.1111/dom.16333 (PMC12046442; doi:10.1111/dom.16333)

**SUPPLEMENTARY MATERIAL**

**Metabolic dysfunction-associated steatotic liver disease, insulin sensitivity and continuous glucose monitoring metrics in patients with type 1 diabetes: a multicenter cross-sectional study**

**Short title:** Insulin sensitivity and MASLD in T1DM

Michela Vergani^1,2^*, Nicolò Diego Borella*^3^, Mariangela Rizzo^1,2^, Matteo Conti^1,2^, Silvia Perra^1^, Eleonora Bianconi^1^, Elena Sani^3^, Alessandro Csermely^3^, Elisabetta Grespan^3^, Giovanni Targher^4,5^, Gianluca Perseghin^1,2^, Alessandro Mantovani^3¶^, Stefano Ciardullo^1,2¶^

^1^Department of Medicine and Rehabilitation, Policlinico di Monza, Monza, Italy
^2^School of Medicine and Surgery, University of Milano Bicocca, Milan, Italy

^3^Section of Endocrinology, Diabetes and Metabolism, Department of Medicine, University and Azienda Ospedaliera Universitaria Integrata of Verona, Verona, Italy

^4^Department of Medicine, University of Verona, Verona, Italy

^5^Metabolic Diseases Research Unit, IRCCS Sacro Cuore-Don Calabria Hospital, Negrar di Valpolicella (VR), Italy

**Supplementary Table 1**. Clinical and biochemical characteristics of adult patients with type 1 diabetes stratified by overweight/obesity status.

|  | **Normal weight (n=96)** | **Overweight (n=67)** | **Obesity (n=35)** | **P values** |
| --- | --- | --- | --- | --- |
| Age (years) | 55±11 | 57±11 | 55±11 | 0.771 |
| Men (%) | 48.9 | 71.6 | 51.4 | **0.012** |
| Diabetes duration (years) | 23.5 (12-36.3) | 28 (18-36) | 35 (22.5-46) | **0.005** |
| Waist circumference (cm) | 83.6±9.2 | 98.0±9.3 | 109.5±11.9 | **<0.001** |
| Systolic blood pressure (mmHg) | 130±19 | 134±16 | 138±22 | 0.059 |
| Diastolic blood pressure (mmHg) | 77±11 | 78±10 | 81±9 | 0.196 |
| HbA1c (%) | 7.5±1.3 | 7.5±0.8 | 7.5±1.2 | 0.934 |
| Total cholesterol (mg/dL) | 164±33 | 155±42 | 160±40 | 0.302 |
| HDL-cholesterol (mg/dL) | 65±15 | 53±11 | 56±15 | **<0.001** |
| Triglycerides (mg/dL) | 69 (29-85) | 82 (63-102) | 72 (64-89) | **0.025** |
| Creatinine (mg/dL) | 0.87±0.30 | 0.85±0.16 | 0.85±0.22 | 0.900 |
| eGFR_CKD-EPI_ (mL/min/1.73 m^2^) | 79±21 | 76±16 | 78±19 | 0.685 |
| AST (IU/L) | 23±7 | 23±6 | 25±9 | 0.761 |
| Liver stiffness (kPa) | 4.6 (3.7-5.4) | 4.7 (3.9-5.7) | 5.8 (4.7-6.9) | **0.001** |
| MASLD (using CAP≥248 dB/m) (%) | 10.4 | 55.2 | 74.3 | **<0.001** |
| MASLD (using CAP≥274 dB/m) (%) | 5.2 | 26.9 | 51.4 | **<0.001** |
| CKD (%) | 12.5 | 16.4 | 25.7 | 0.191 |
| Microalbuminuria (%) | 6.4 | 9.2 | 11.8 | 0.587 |
| Macroalbuminuria (%) | 4.3 | 1.5 | 0 | 0.329 |
| Diabetic retinopathy (%) | 30.2 | 35.8 | 48.6 | 0.150 |
| Hypertension (%) | 42.7 | 46.3 | 48.8 | 0.807 |
| Ischemic heart disease (%) | 2.1 | 7.5 | 5.7 | 0.251 |
| Ischemic stroke (%) | 1.04 | 1.5 | 0 | 0.773 |
| Daily insulin dose (IU/Kg/day) | 0.5±0.2 | 0.6±0.3 | 0.7±0.3 | **<0.005** |
| Metformin (%) | 8.3 | 7.5 | 20.0 | 0.099 |
| Anti-platelets (%) | 15.6 | 25.4 | 22.9 | 0.285 |
| ACE-inhibitors/ARBs (%) | 29.2 | 31.3 | 37.1 | 0.684 |
| Beta blockers (%) | 10.4 | 10.5 | 14.3 | 0.805 |
| Calcium antagonists (%) | 11.5 | 14.9 | 11.4 | 0.785 |
| Diuretics (%) | 10.4 | 8.9 | 5.7 | 0.708 |
| Statins (%) | 44.8 | 59.7 | 51.4 | 0.173 |
| *CGM metrics* | | | |  |
| TIR 70-180 mg/dL | 58.6±17.8 | 59.7±14.9 | 58.6±15.4 | **0.025** |
| TBR | 2.7±3.3 | 2.9±3.4 | 3.1±3.4 | 0.842 |
| TBR1 | 2.4±2.6 | 2.6±2.9 | 2.5±2.7 | 0.897 |
| TBR2 | 0.3±0.8 | 0.3±0.6 | 0.4±0.8 | 0.533 |
| TAR | 38.7±18.8 | 37.3±15.2 | 36.5±16.7 | 0.856 |
| TAR1 | 24.7±9.5 | 24.6±9.2 | 25.8±8.2 | 0.699 |
| TAR2 | 13.4±13.0 | 10.9±8.8 | 11.5±9.5 | 0.808 |
| GRI | 47.7±23.2 | 44.3±17.5 | 46.2±18.6 | 0.722 |
| GRI hypoglycemia component | 6.5±8.1 | 7.2±8.3 | 7.2±8.3 | 0.835 |
| GRI hyperglycemia component | 41.2±24.5 | 37.1±16.3 | 38.9±19.4 | 0.733 |
| %CV | 35.9±5.0 | 35.9±5.1 | 35.9±4.9 | 0.957 |

Sample size, n=198. Data are expressed as means ± SD, medians, and interquartile ranges (IQRs) or percentages. Differences among the two patient groups were tested by the Chi-squared test for categorical variables, the Student’s t test for normally distributed continuous variables, and the Mann-Whitney U test for non-normally distributed variables (i.e., diabetes duration, triglycerides, AST, liver stiffness measurement).

CKD was defined as eGFR_CKD-EPI_ <60 mL/min/1.73 m^2^ and/or macroalbuminuria.

*Abbreviations*: ACE, angiotensin-converting-enzyme inhibitor; ARB, angiotensin II receptor blocker; AST, aspartate aminotransferase; BMI, body mass index; CGM, continuous glucose monitoring; CKD, chronic kidney disease; eGFR_CKD-EPI_, estimated glomerular filtration rate calculated by the CKD-Epidemiology Collaboration study equation. CV, Coefficient of variation; GRI, Glycemia Risk Index; MASLD, metabolic dysfunction-associated steatotic liver disease; TAR, Time above range >180 mg/dL (>10.1 mmol/L); TAR1, Time above range 181-250 mg/dL (10.1-13.9 mmol/L) (high glucose or Level 1 hyperglycemia); TAR2, Time above range >250 mg/dL (>13.9 mmol/L) (very high glucose or Level 2 hyperglycemia); TIR, Time in range 70–180 mg/dL (3.9–10.0 mmol/L); TBR, Time below range <70 mg/dL (< 3.9 mmol/L); TBR1, Time below range 54-69 mg/dL (3.0 - 3.9 mmol/L) (low glucose or Level 1 hypoglycemia); TBR2, Time below range <54 mg/dL (<3.0 mmol/L) (very low glucose or Level 2 hypoglycemia)

**Supplementary Table 2**. Univariable and multivariable linear regression analyses – Association between estimated glucose disposal rate (eGDR) and MASLD, using a CAP threshold of 274 dB/m, in adult patients with type 1 diabetes.

|  | **Beta coefficients** | **95% Confidence Intervals** | **P values** |
| --- | --- | --- | --- |
| **Unadjusted model** |  |  |  |
| MASLD (yes *vs.* no) | -0.348 | -0.479 to -0.218 | **<0.001** |
|  |  |  |  |
| **Adjusted model 1** |  |  |  |
| MASLD (yes *vs.* no) | -0.136 | -0.258 to -0.012 | **0.031** |
| Age (years) | -0.011 | -0.016 to -0.007 | **<0.001** |
| Sex (men *vs.* women) | -0.066 | -0.166 to 0.033 | 0.191 |
| Body mass index (kg/m^2^) | -0.017 | -0.025 to -0.008 | **<0.001** |
| Triglycerides (mg/dL) (Log Scale) | 0.009 | -0.020 to 0.197 | 0.108 |
| Time above range (181-250 mg/dL) (TAR) (Log Scale) | -0.069 | -0.147 to 0.009 | 0.083 |
| Diabetes duration (years) (Log Scale) | -0.001 | -0.004 to 0.003 | 0.845 |
| Daily insulin dose (IU/Kg/day) | -0.473 | -0.682 to -0.264 | **<0.001** |
| CKD (yes *vs.* no) | -0.015 | -0.149 to 0.118 | 0.820 |
| Liver stiffness (kPa) (Log Scale) | -0.001 | -0.022 to 0.022 | 0.986 |

Sample size, n=198. Data are expressed as beta coefficients and 95% confidence intervals estimated by linear regression analyses. The dependent variable of all models was the estimated glucose disposal rate (eGDR) (that was logarithmically transformed before the analyses). MASLD (metabolic dysfunction-associated steatotic liver disease) was defined as CAP ≥274 dB/m and presence of a cardiometabolic risk factor. CKD (chronic kidney disease) was defined as eGFR_CKD-EPI_ <60 mL/min/1.73 m^2^ and/or macroalbuminuria.

**Supplementary Figure 1.** Boxplots of the estimated glucose disposal rate (eGDR) in adult patients with T1DM stratified by MASLD status, evaluated by vibration-controlled transient elastography (VCTE) with controlled attenuation parameter (using a CAP threshold of 274 dB/m).


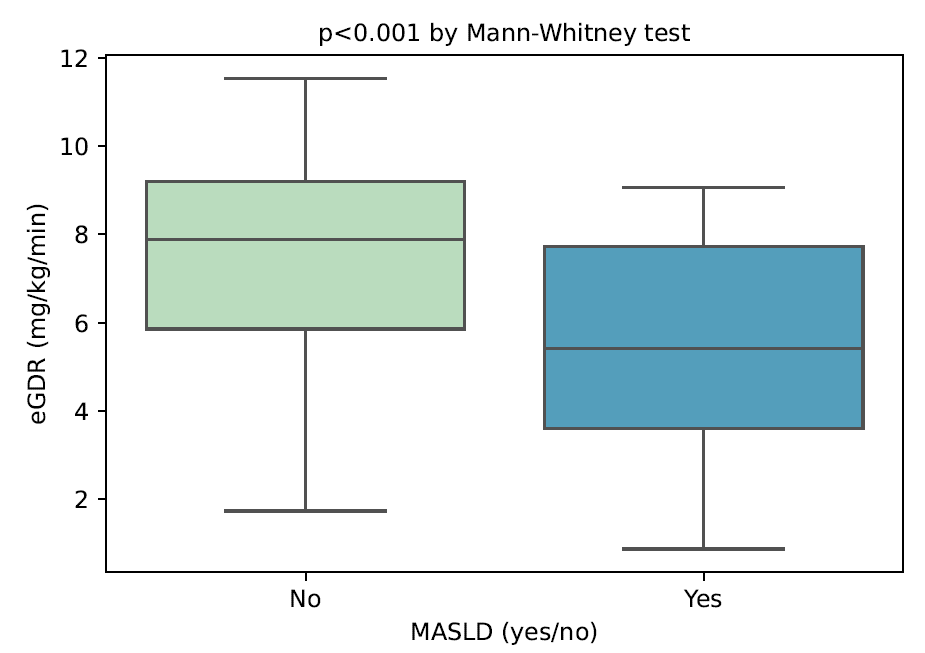


**Supplementary Figure 2.** Percentages of daily insulin dose tertiles by MASLD status, diagnosed using either a CAP threshold of 248 dB/m (Panel A) or a CAP threshold of 274 dB/m (Panel B).


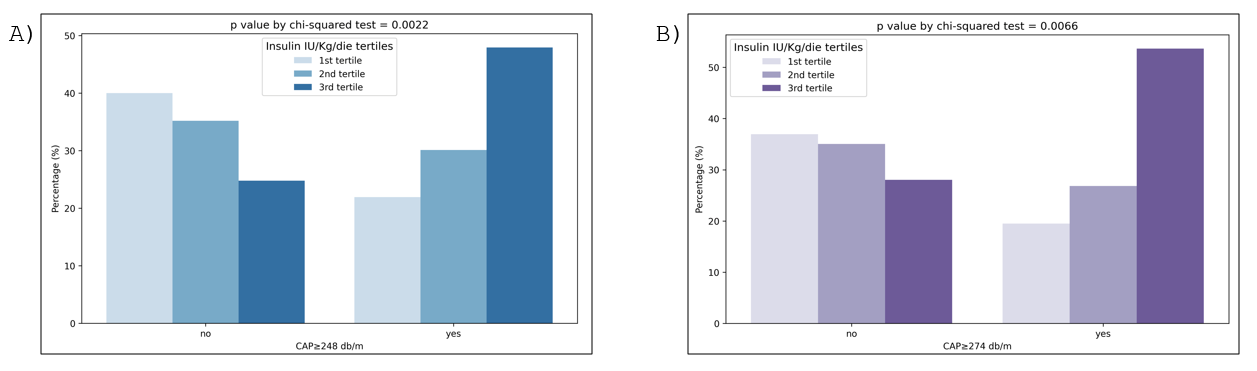

Supplement: Supplementary file 1 — FIGURE S1. Boxplots of the estimated glucose disposal rate (eGDR) in adult patients with T1DM stratified by MASLD status, evaluated by vibration‐controlled transient elastography (VCTE) with controlled attenuation parameter (using a CAP threshold of 274 dB/m). FIGURE S2. Percentages of daily insulin dose tertiles by MASLD status, diagnosed using either a CAP threshold of 248 dB/m (Panel A) or a CAP threshold of 274 dB/m (Panel B). TABLE S1. Clinical and biochemical characteristics of adult patients with type 1 diabetes stratified by overweight/obesity status. TABLE S2. Univariable and multivariable linear regression analyses – Association between estimated glucose disposal rate (eGDR) and MASLD, using a CAP threshold of 274 dB/m, in adult patients with type 1 diabetes. [file DOM-27-3201-s001.docx]
